# Background, Applications and Issues of the Experimental Designs for Mixture in the Food Sector

**DOI:** 10.3390/foods10051128

**Published:** 2021-05-19

**Authors:** Giacomo Squeo, Davide De Angelis, Riccardo Leardi, Carmine Summo, Francesco Caponio

**Affiliations:** 1 Department of Soil, Plant and Food Science (DISSPA), University of Bari Aldo Moro, Via Amendola 165/a, 70126 Bari, Italy; davide.deangelis@uniba.it (D.D.A.); carmine.summo@uniba.it (C.S.); francesco.caponio@uniba.it (F.C.); 2 Department of Pharmacy (DIFAR), University of Genova, Viale Cembrano 4, 16148 Genova, Italy; riclea@difar.unige.it

**Keywords:** food formulation, ingredients, product development, DoE, optimization, food quality, food modelling

## Abstract

Background: Mixtures play a key role in Food Science and Technology. For studying them, rational approaches should be used. In detail, the experimental designs for mixtures are useful tools for studying the effects of ingredients/components in formulations. Results: Food Science and Technology is the fourth category among the total records considered in this review. The applications span from food formulation to the composition of modified atmosphere, shelf-life improvement and bioactives extraction. However, the majority of the studies regards few products and ingredients. Simplex-lattice and simplex-centroid designs are the most common used, although some optimal designs, such as the D-optimal, have also interesting applications. Finally, some issues are highlighted, which basically regard the interpretation of the models coefficients and the lack of model validation. Conclusion: In the last decade, mixture designs have been fairly used in the field of Food Science and Technology. Modeling the response(s) allows researchers to achieve a global knowledge of the system under study within the defined experimental domain. However, the majority of application has regarded limited classes of products, and thus an increase in the spectrum of applications is desired.

## 1. Introduction

Mixtures are common matter of everyday life and of many fields of science. Actually, it is quite difficult to think at situations in which mixtures are not concerned. The meaning of “mixture” could be slightly different considering the context as well as different could be the way in which mixtures are prepared. Further, according to the complexity, we can have binary mixtures (made up of two components), ternary mixtures and so on [1,2,3,4,5].

Mixtures are particularly important for the food sector in which, for instance, the formulation of a product consists in the definition of the ingredients and, most important, of their relative proportion in the mixture [6,7]. The concept of “proportions” is a key concept which makes the mixture problem so special [8]. Common situations easily found in the food sector could be the composition of gases for a modified atmosphere packaging [9], a blend of olive oils or wines [10], the composition of a liquid medium for food storage, etc. According to the final aim, food scientists and technicians might be interested in studying and optimizing their mixture for (i) technological applications (e.g., increasing products shelf-life); (ii) nutritional targets (e.g., reaching the required content of fiber to ensure the compliance with health claims); and (iii) organoleptic scopes (e.g., obtaining the desired textural and sensory properties of a baked good).

Considering their widespread diffusion, the study of mixture is of fundamental importance. There are rational approaches that can be used to study the mixtures and they are basically included in the field of the design of experiments (DoE) [8,11]. DoE generally refers to a collection of tools aimed at systematically examining different types of problems in order to obtain the best compromise between information and effort (time, money), i.e., with a relatively small number of experiments [7,12]. DoE is part of the vast area of chemometrics [13].

According to Hibbert (2016)[14], the experimental design is an “*efficient procedure for planning combinations of values of factors in experiments so that the data obtained can be analyzed to yield valid and objective conclusions*”. To reach such important goal (valid and objective conclusions), there are key steps that could be summarized in the following [11]: (i) define the goal of the experiments; (ii) detect all the factors that can have an effect; (iii) plan the experiments; (iv) perform the experiments; and (v) analyze the data obtained by the experiments.

It should be understood that in this process, the critical step is the planning of the experiments. Once these have been planned in a rational way (which means answering the question: which combinations of the selected factors should I test to gain the maximum information about my system?) they can be performed. Then, regression and analysis of variance (ANOVA) are the tools extensively used to find the relationship between the dependent variables (the response(s) of interest) and the independent variables (the factors) and to evaluate the significance of the factors [12]. More detailed information about the theory and use of DoE can be found elsewhere [8,11,13,15].

Inside the areas of DoE, a special case is represented by the experimental designs for mixtures. Indeed, mixtures have some particularities which make such designs different from the others. In the light of these considerations, the aim of this work is to review the usage of these tools in the field of Food Science and Technology with the scope to highlight the potentialities of such approach. Before doing this, a brief recap of the mixture problem, the models and the designs that can be used is reported.

### 1.1. The Mixture Context

In the next paragraphs, it is our aim to recall some basic information about the mixture problem and the relative experimental designs that may help those readers who are not familiar with the topic. The readers interested in learning more about the topic may refer to other works [7,8,11,13,15].

As already stated, a mixture could be defined in several ways. It could be defined as a “*portion of matter consisting of two or more chemical substances called constituents*” [16] or, more generally, as a “*substance made from a combination of different substances, or any combination of different things*” [17]. Further, most important to our scope, by the chemometric point of view, it could be considered a combination of factors (i.e., ingredients) whose total is a constant value, 1% or 100%.

**Figure 1:**
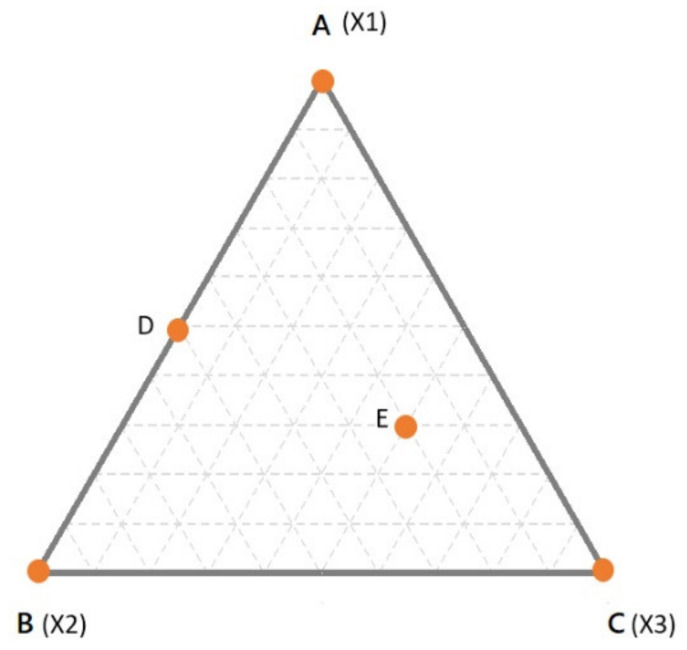
A simplex for a ternary mixture. Dashed lines indicate intervals of 10% of the components.

Before proceeding, it would be useful at this moment to recall how mixtures are represented. This is done by the so-called “*simplex*”, which is the simplest object in the space of a defined dimensionality (*n*, equal to the number of components of the mixture) having *n*-1 dimension(s) [7,13]. For example, for a binary mixture, the simplex is a line in one dimension; for ternary mixtures, the simplex is a triangle in two dimensions; for a quaternary mixture, the simplex is a tetrahedron in 3-dimensions. In Figure 1, the simplex for a three components mixture is reported. In the simplex, the vertices correspond to the pure components (i.e., 100% of X1, X2, X3—points A, B and C respectively). The edges of the simplex correspond to all the binary mixtures made up of the components at the vertices of the edge under consideration while the component at the opposite vertex is absent (e.g., point D). Finally, any point inside the simplex represents a ternary mixture (e.g., point E). In Figure 1, the proportion of each component in all the possible binary or ternary mixtures is simply obtained by drawing lines parallel to the edge opposite to the vertex of interest. For example, considering the binary mixture in D, the proportion of X1 can be found on the line parallel to the edge X2–X3 (which is opposite to the vertex X1), which passes through D. In this case, D being a binary mixture between X1 and X2, it is easy to conclude that the relative amount of X1 is 50% (or 0.5).

However, in case of doubts, it may help to simply remind that the vertex represents 100% of that component. Thus, it is obvious that the closer a point is to a specific vertex, the higher is the relative amount. To make it clearer, let us consider the point E. As always, the proportions of the components are found moving in lines parallel to the edge opposite to the corresponding vertex. The line passing through E, which is parallel to X2–X3 edge, tells us the proportion of X1. However, one may be confused if X1 is 0.3 or 0.7. Considering that the line is quite far from the X1 vertex and next to the X2–X3 edge (which is 0% of X1) it is clear that the relative amount should be 0.3. The same goes for the other components and it comes out that the mixture in E is made up of 30% of X1, 20% of X2 and 50% of X3. The sum, of course, is 100%. The interpretation becomes more complicated when the dimensions are higher than 3.

Having in mind the simplex in Figure 1, some peculiarities of the mixture designs (MD) could be highlighted. First of all, it could be easily verified that for each point in the simplex, the implicit constraint (total sum equals to 100%) is fulfilled. Given this implicit dependence, the natural consequence is that the factors of a mixture are no longer independent [11,15]. For independence we intend the possibility to vary the level of a factor independently from the other factor(s) [11,18]. In the mixture context, increasing the amount of X1 will necessarily cause modification in the other component(s). Due to this dependence, the designs employed in other contexts (such as factorial designs), for which the independence of the factors is a fundamental requirement, cannot be applied.

A second important consideration is that mixture designs are thought to study the effect of variation in the proportions of ingredients instead of variation in their absolute values [6,8,11]. In other words, if we are studying the effect of a mixture of N_2_, CO_2_ and O_2_ on the shelf-life of a packaged food or the effect of different flours on dough properties, it does not matter if the package headspace is 10 mL or 100 mL, or the final dough weight is 100 g or 10 kg. What we are looking for is, for instance, the effect of changing the amount of CO_2_ from 20% to 40% (and obviously reduce the relative amount of the other components).

**Figure 2:**
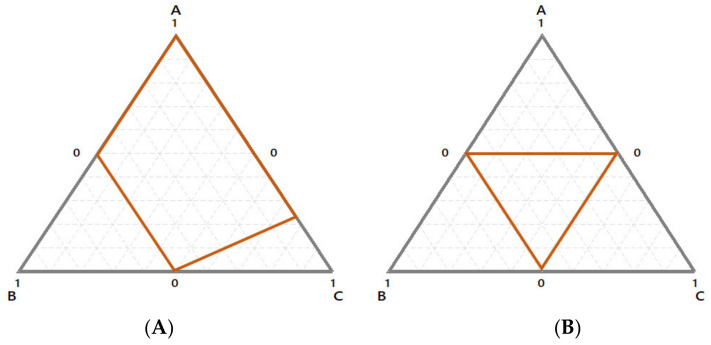
Reduced domains with irregular (**A**) or regular shape (**B**).

Until now, we have seen that variables in mixture problems are proportions and their sums must match the total. However, in a real-life scenario, quite often additional constraints should be considered. Indeed, it may happen that the proportion of a component cannot span the whole range, i.e., from 0% to 100% [19,20]. In these cases, a suitable range of the component(s) must be fixed. As a result, the experimental domain is reduced, and the final shape could be different, as reported in Figure 2.

In the first case (Figure 2A) the constrains define a subspace with irregular domain, whereas this is not true for the second one (Figure 2B).

By the practical point of view, when possible, it could be advisable to reduce the space to a regular form such as that in Figure 2B in order to apply the standard designs (see next paragraph). However, when it is not possible (Figure 2A), the best experimental points could be selected by approaches such as the D-optimal criterion [11,21,22,23]. When the simplex is reduced, components could be transformed in the so-called *pseudo-components*. Ways for transformation in pseudo-components are reported elsewhere [7,8,13].

### 1.2. Designs for Mixtures

Before a brief overview about the available designs for mixtures, it is worth recalling that a mathematical model is associated to each experimental design (not only for mixtures) [7]. Actually, the design is only the tool to calculate the model which is the real information we are looking for. The model should be a priori defined by the investigator according to her/his aims and it defines the postulated relationship between the factors and the response(s). The models for MD are generally presented in the so-called canonical form [8] and the most common ones are the linear (Equation (1)), the quadratic (Equation (2)) and the special cubic (Equation (3)).

(1) $$y=b_{1}X_{1}+b_{2}X_{2}+b_{3}X_{3}$$

(2) $$y=b_{1}X_{1}+b_{2}X_{2}+b_{3}X_{3}+b_{12}X_{1}X_{2}+b_{13}X_{1}X_{3}+b_{23}X_{2}X_{3}$$

(3) $$\begin{array}{l} y=b_{1}X_{1}+b_{2}X_{2}+b_{3}X_{3}+b_{12}X_{1}X_{2}+b_{13}X_{1}X_{3}+b_{23}X_{2}X_{3}\\ \;\;\;\;\;\;\;+b_{123}X_{1}X_{2}X_{3} \end{array}$$

where y, indicate the response(s); $b_{1},\;b_{2}\;\mathrm{and}\;b_{3}$ indicate the linear terms coefficients; $b_{12},\;b_{13}\;\mathrm{and}\;b_{23}$ indicate the coefficients of the two-interaction terms; $b_{123}$ indicates the coefficient of the three-interaction term; and $X_{1},X_{2}\;\mathrm{and}\;X_{3}$ indicate the components under study.

It is worth highlighting that in the canonical forms of the quadratic and special cubic models, the quadratic terms (i.e., x^2^) and the cubic terms (i.e., x^3^) are not present. Indeed, the quadratic and the special cubic models allow to estimate the linear and all the two-interaction terms and the linear, the two- and three-interaction terms, respectively.

In this kind of models, the coefficients of the linear terms represent the response obtained solely with the *i*th pure component, while the interaction terms show the deviation from the sole additive effect of the components (i.e., positive or negative synergic effects) [6,11].

There are two well-known kinds of designs for mixtures: (i) the simplex-lattice designs and (ii) the simplex-centroid designs [8,13]. In the simplex-lattice, the levels of each component are defined by the ratios *r/m* (where *m* is the polynomial degree to be fitted and *r* goes from 0 to *m*) and the experimental points are the combinations of these levels. For a quadratic model (*m* = 2) with three components, the levels of each component are 0 (0/2), 0.5 (1/2) and 1 (2/2) and the experimental points are (1,0,0), (0,1,0), (0,0,1), (0.5,0.5,0), (0.5,0,0.5) and (0,0.5,0.5). By contrast, in simplex-centroid designs the experimental levels are defined by the ratios 1/*q* for *q* going from 1 up to the number of components. With *q* = 3 we have 1 (1/1), 0.5 (1/2), 1/3 and, of course, 0. Then, the experimental points are all the combinations of these levels for a total number of equals to 2*^q^* − 1 [13]. The experimental points for a special cubic model with a ternary mixture are reported in Table 1.

In simple-lattice designs, the factor levels depend on the postulated model regardless the number of components and blends of *m*-components at a time are tested (being *m* the model degree). This means that for modeling a quadratic model, blends of two components per time will be considered whether there are three or five components [15]. The number of experiments varies according to the postulated model (Table 2).

Differently, simplex-centroid designs are reported to be model independent, being dependent only on the number of components, according to the formula 2*^q^* − 1 (Table 2) [13]. However, in practice, it could be argued that also in the case of simplex-centroids the experimental points are related to the postulated models and not only on the formula 2*^q^* − 1. A clear example is reported in [6], where the authors started by postulating a simple model (i.e., linear) which could be calculated using only the three experiments at the vertices of the simplex. Then, the experimental blends at the edges are used for validation. If the linear model is rejected, the edge experiments are used to calculate the higher model (i.e., quadratic). This example shows that the minimum number of experiments required by a simplex-centroid design could be lower than expected by the application of the rigid formula and could vary according to the postulated model.

Moreover, the so-called augmented designs could be found which consider other experiments generally located at half-way between each vertex and the center (i.e., blends made up of 2/3, 1/6 and 1/6 in a ternary mixture) [24,25,26,27].

After the experiments have been performed, the response(s) could be modeled, and the model coefficients could be estimated, together with their significance and the figures of merit of the regression [12,15]. By the practical point of view, it should be considered that the interpretation of the model coefficients in mixture designs is not so straightforward such as that for designs for independent variables, because the coefficients are not related to the effects [8,11,13]. Thus, a better and much more reliable interpretation of the components effect on the response(s) could be obtained by looking at the response surfaces [11,28].

Finally, model validation should be always carried out [6,11,12].

## 2. Materials and Methods

This review is based on a literature search carried out through the ISI-Web of Science Core Collection (SCI), considering all the research works published in a 10-year range, from 2011 to present (18 February 2021). The keywords used for the bibliographic survey were “*mixture-design*”, “*design of experiment* AND *mixture*”, “*Experimental design* AND *mixture*”, “*DoE* AND *mixture*”, “*Formulation* AND *Design*” in both the topic (searches title, abstract, author keywords, and Keywords Plus) and the title.

With the aim of giving to the reader a general overview of the literature search results, the number of papers in which the keyword “mixture-design” was mentioned in the topic are reported in Table 3, and the results were classified according to the Web of Science Categories.

Considering the number of results recorded in the topic search for the keyword “mixture-design”, it is possible to highlight the predominance of the mixture design in the materials science and in the engineering area. Food Science and Technology is the fourth category in which the mixture design is used, followed by the pharmaceutical science.

Moreover, to better refine the research, the investigation was focused on the papers belonging to the Food Science and Technology category which had the keyword “*mixture-design*” within the title. The keyword was chosen as it is very specific and precise according to the aims of this review. From this further research, 106 papers were identified and then studied and discussed in the next section.

## 3. Results

**Figure 3:**
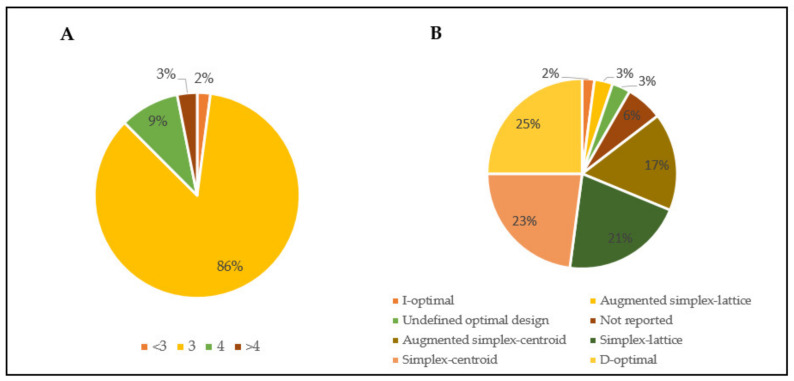
(**A**) Mixture complexity and (**B**) type of designs in the surveyed articles, respectively (*n* = 96).

This part is aimed to show the versatility of the experimental designs for mixtures in the food sectors which cover a wide range of applications. Some of the results (ten) have been excluded because out of the scope. On the whole, the majority of applications consider ternary mixtures (Figure 3A). In a couple of cases binary mixtures were studied and only in one case were five and six components considered. As already reported, studying mixtures in which components are >3 becomes more complicate starting from the limitations in the experimental space visualization and, consequently, in model interpretation [11]. To overcome this issue, an interesting approach could be to split the whole mixture in subdomains each made up of an acceptable number of components [29].

The MD used in the food sector are mostly the canonical ones (simplex-lattice and simplex-centroid) (Figure 3B).

In addition, optimal designs are rightly used when restricted domains do not allow to use the classic ones (see Section 1.1). Among these, D-optimal is the most commonly used, although some examples of I-optimal are found, too.

**Figure 4:**
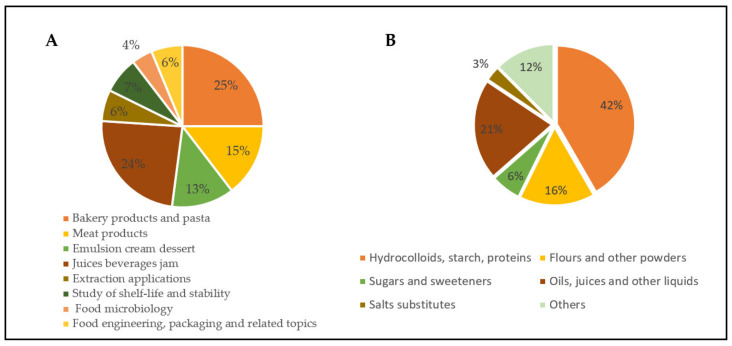
(**A**) Food products/applications and (**B**) type of components in the surveyed articles, respectively (*n* = 96).

Considering the applications, it could be observed that MD in food science in the last decade have been mostly used for bakery/pasta and for juices/beverages/jams. Together, these two categories cover roughly 50% of the total applications (Figure 4A). Accordingly, the main components studied were hydrocolloids/starch/proteins which alone cover almost half of the surveyed applications (Figure 4B). Hydrocolloids are widely used in food formulation thanks to their functional properties. In processed foods they are almost ubiquitous [30], and the reported results confirm the great interest toward their use. Other remarkable applications regard meat products and emulsions/creams/desserts (Figure 4A). On the contrary, a less usage of MD has been recorded for extraction studies, food microbiology and food engineering. It is in those areas that efforts should be made to improve the knowledge about the advantages of the use of MD approaches. For example, recovery of bioactives from foods or food by-products has become an hot topic in recent years [31,32,33] but, despite this interest, in our research only few works have been found that optimize the extraction mixtures according to MD.

Looking at the objectives, the majority of the research articles studied the textural, the rheological and/or the sensorial properties of foods as affected by the formulation. Food texture is one of the main parameters, together with the sensory quality, that strongly influences the consumer decisions and the willing to buy a certain product. Therefore, in this perspective, MD can strongly help new product development and optimization.

**Figure 5:**
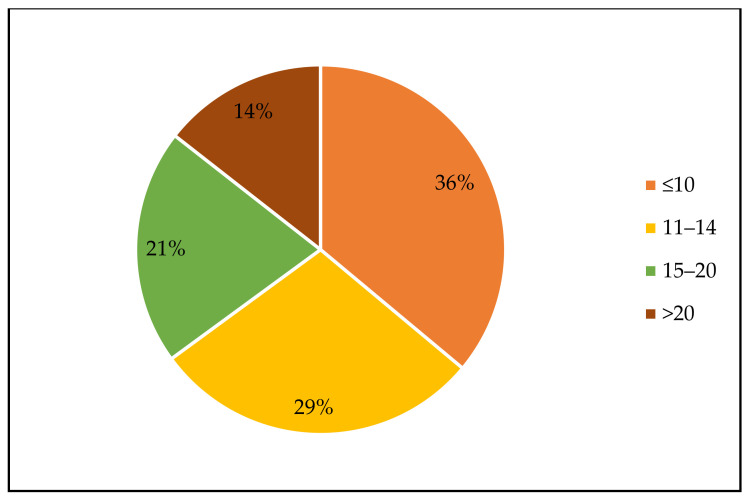
Number of experiments carried out in the surveyed articles (*n* = 96).

Finally, following DoE approaches has the important advantage to reduce the number of experiments—and thus the efforts—giving, at the same time, information of a higher quality. From the literature overview, it emerged that only in few cases the number of experiments exceed 20, and in 35 out of 96 papers the number was lower or equal than 10 (Figure 5). In Table 4, a synoptic scheme of the surveyed literature together with the main features of the employed designs and their statistical evaluation is reported, while the succeeding paragraphs give a brief presentation of the surveyed works.

### 3.1. Study and Optimization of Product Formulation

#### 3.1.1. Case Studies on Bakery Products and Pasta

Bread and other bakery products are continuously investigated by researchers in order to improve their nutritional quality or to design products for special consumers (e.g., gluten-free, rich in protein, fat-reduced).

Encina-Zelada et al. [34] studied the influence of hydrocolloids for the formulation of gluten-free bread with the aim to study the possibility of synergistic effect through the combination of xanthan gum (XG), hydroxypropyl methyl cellulose (HPMC) and guar gum (GG). The D-optimal mixture design consisted in 11 experiments and the total amount of hydrocolloids reached the 4% of the formulation. The quality parameters of the bread were analyzed by a two-factor interaction polynomial linear model. The authors found significant interactions between XG and GG, whereas HPMC interacted with GG and they improved some physicochemical properties of bread, such as the specific volume, the softness and the porosity of the crumb. Finally, an optimization study was carried out to maximize the specific volume of the crumb, the brown index of the crust, as well as minimize the hardness and the cell density of the crumb.

The bread quality parameters can be optimized in glute-free formulation also by investigating the effect of the main ingredients such as rice flour, maize starch and wheat starch, as reported in Mancebo et al. [35]. The mixture design proposed by the authors was a simplex centroid performed in duplicate, and the responses were not only instrumental (e.g., texture profile, image and color analysis) but also the overall acceptability was considered. After the modeling with a special cubic model, the multiple response method was used to optimize the formulation, as function of both instrumental and sensory analysis.

So far, the mixture designs analyzed concerned the study of three ingredients. However, mixture design can be easily applied to more components with different strategies of design. For example, Rößle et al. [36] designed a D-optimal experiment at four components (inulin, oligofructose, margarine and sugar) for the production of quick breads. In particular, the sum of four ingredients reached the 20% of the formulation, whereas the remaining 80% was constituted by the other ingredients. With a total of 24 experiments, the authors focused on the possibility to use inulin and oligofructose as fat and sugar substitutes, analyzing as responses, the usual quality parameters seen for the previous articles (texture, crumb porosity, color indices, bread volume). The responses were modeled by a special cubic model which was then validated. The authors obtained a good prediction level for the optimized formulations and suggested that future studies will be focused on the nutritional and sensory aspects since the technological quality was already optimized.

In another work carried out by Santos et al. [37] the ingredients studied through the mixture design were five, i.e., chickpea flour, cassava starch, maize starch, potato starch and rice flour. The authors decided to prepare six different simplex-centroid designs at three components with three factors (1, 1/2, 1/3) in order to identify the optimal ratios of the chickpea flour with the different blends of the other ingredients. The physical properties of the bread were evaluated and, as in the previous studies, the optimal formulations were identified.

Besides bread, mixture design was widely applied also to other bakery products such as cakes and baked goods [38,39,40,41,42] with different purposes.

Masmoudi et al. [40] investigated the addition of hydrocolloids (xanthan gum, carboxymethyl cellulose and κ-carrageenan) with the aim of solely improve the textural quality of acorn muffin in order to have a close structure to the conventional muffin produced with wheat flour. The augmented simplex centroid design with 10 experiments was used, and the optimal formulation was only predicted but not produced. In this case as well, the authors suggest that further study will involve the optimization of the sensory properties.

Both texture and sensory quality of gluten-free cakes were optimized by adding protein ingredients [38,41]. Ammar et al. [38] studied the influence of maize and rice flours (0–100%) in combination with whey protein concentrate (0–15%) in a gluten-free sponge cake. Bravo-Nunez et al. [41] analyzed the effect of pea, whey and egg white proteins in gluten-free layer cake by using a simplex lattice design with 10 experiments. Interestingly, Ammar et al. [38] carried out the optimization by giving higher importance to different criteria: (i) the cost of the product and the energy value, (ii) the physicochemical properties. Then, the two selected formulation were analyzed for the microstructural and the sensory properties. Finally, the authors concluded that the incorporation of the 6.5% of whey protein concentrate led to the best results.

The mixture design was also applied to pasta, with the aims of study the rheological properties of a gluten-free dough [20], optimize the tensile strength of rice noodles [43] or formulate a protein-fortified pasta for athletes [44].

Larrosa et al. [20] while preparing their simplex centroid design pointed out the importance of the constrains coming from the preliminary trials. In particular, they stated that was not possible to laminate the pasta dough outside the range of the water content between 35.5% and 39.5%, therefore, the experimental domain was adjusted according to the requirement of the food. The rheological properties were modeled with a second order polynomial model, furthermore, the optimization was carried out by the desirability function.

Kamali Rousta et al. [44] study the formulation of athletic pasta, prepared a D-optimal design with all the six ingredients of the product (semolina, pea protein isolate, whey protein isolate, soy protein isolate, oat flour and gluten), leading to 31 experimental trials. The usual linear, quadratic and special cubic models were fitted to each of the responses (hardness, protein). The optimization was carried out reaching the best compromise between the protein content and the hardness (according to the desirability function) and just one formulation was proposed and subjected to the sensory evaluation.

#### 3.1.2. Case Studies on Meat and Meat Products

Mixture design has been widely applied to meat and meat products to (i) optimize the fat reduction [56,57,58]; (ii) improve the nutritional quality by adding prebiotic ingredients [59,60], bioactive compounds [61], salt substitutes [62] and supplementary sources of protein [63].

Afshari et al. [56] evaluated the effect of a mix composed of breadcrumb, inulin and beta glucan on the physicochemical properties and the overall acceptability of beef burgers, produced following a three-factor at six-levels mixture design. The sum of the three components was the 8% of the total and 12 experiments plus two replicates were performed. The linear, the quadratic and the special cubic model were then fitted to each of the responses analyzed. The results indicated the interaction between inulin and beta glucan which improved the physicochemical properties of the burgers without damaging the sensory quality. Finally, the optimization step allowed to the definition of the best ratios between the three ingredients.

The addition of prebiotic ingredients in meat products was also studied by Amini Sarteshnizi et al. [60] who optimized the formulation of prebiotic sausages supplemented with resistant starch (RS), beta glucan (BG) and starch (ST). The ingredients constituted the 6% of the total formulation and they were combined according to a D-optimal mixture design consisting in eight experiments plus five replicates. In this study as well, the physicochemical and sensory properties were fitted to a linear, quadratic, or special cubic models. The authors pointed out that the RS alone was not suitable in the formulation since its negative impact on the physicochemical properties and the sensory acceptability, whereas the combination between RS and BG led to a soft texture. The optimum formulation was also identified through the desirability function and it contained 2.2% RS, 1.3% BG and 2.5% ST.

Baugreet et al. [63] used a simplex centroid D-optimal mixture design with constrains to study the effect of meat content (90–100%), rice protein (0–10%) and lentil flour (0–10%) on physicochemical, microbiological and sensory quality of beef patties, specifically designed for an older adult diet. The model consisted of seven experimental points plus 10 replicates and the results were fitted to linear, quadratic and special cubic models according to the significance, the coefficients of determination and the lack of fit. The model was also validated calculating the accuracy and the bias factors. The optimization study showed that the addition of small quantity of rice protein (1–4%) and lentil flour (4–7%) improved not only the protein content but also the texture and the sensory quality of beef patties.

Marchetti et al. [62] optimized the reduction of the sodium content in lean sausages by using a simplex-lattice mixture design. The proportion of the three ingredients were constrained, setting the ranges of NaCl, KCl and sodium tripolyphosphate (TPP) to 0.5–1.4, 0–0.7 and 0–0.5 g/100 g, respectively. The ranges were identified according to previous studies that reported the maximum level of usages for KCl and TPP. This highlights that the mixture design can be easily adaptable also to a very small quantity of the ingredients. The design consisted in 11 experiments with three replications of the centroid point. The experimental data were fitted to a cubic model and then validated. According to the physicochemical and the textural properties, the salt substitutes content was optimized through the desirability function. The authors decided to carry out the optimization prior the sensory analysis which was performed only on the optimized formulation and on the control. Despite the alteration of the structure given by the salt substitution, the sensory properties were not affected.

The improvement of the nutritional value of meat products can be achieved also by the addition of bioactive compounds, as reported by Nurain et al. [61]. The authors used a simplex-lattice mixture design to investigate the effect of three natural antioxidants from aromatic herbs in chicken sausages. The design consisted in 10 experiments plus three replicates and the results of the total phenolic compounds, antioxidant activity and sensory analysis were fitted by linear, quadratic or special cubic models. Once identified, the optimal region according to the target, the model was validated and the *t*-test was used to determine significant differences between the predicted results and actual values of the optimized formulae.

#### 3.1.3. Case Studies on Emulsions, Creams, Dessert

Emulsions, creams, yogurt and desserts obtained from different sources (both animal or plant) have been the focus of several optimization efforts in order to reduce the fat content, substituting ingredients and obtain improved products with physicochemical properties similar to the conventional ones.

In the work of Nikzade et al. [69] a simplex-centroid mixture design was used to optimize the proportions of three components (xanthan gum, guar gum, and mono- and diglycerides emulsifier) in a mayonnaise-like formulation. A total of 10 experiments were carried out, seven of which are the typical experiments of a simplex-centroid (Table 1) plus the augmented blends at the levels 2/3, 1/6 and 1/6. The physicochemical and textural properties as well as the overall acceptance were modeled according to linear, quadratic and special cubic models. Although no validation was carried out, the best combinations to produce low-cholesterol and low-fat emulsions with soy milk close to the commercial mayonnaise, was predicted. The reduction of fat in mayonnaise was also studied by Rahbari et al. [70]. A ternary mixture composed of wheat germ protein isolate (WGPI), xanthan gum and egg yolk (EY) was studied. Giving the defined constrains which result in an irregular domain, the 10 experimental points were chosen according to an optimization criterion which was not reported by the authors. The responses were the physicochemical properties, the texture and overall acceptance which were modeled according to the linear, quadratic and special cubic models. The contour plots of the responses were then used to define a suitable proportion of the components which was found to be 7.87 g, 0.2 g and 0.93 g of WGPI, XG and EY, respectively.

Polysaccharides were used in diary and low-fat dairy desserts to improve the rheological and sensorial properties [71,72]. For example, Toker et al. [71] studied the effect of carrageenan, alginate, guar and xanthan gums by a simplex-lattice mixture design. A cubic model was postulated (20 experiments for a quaternary mixture—Table 2) to which the authors added further experiments, plus some replicates up to a final number of 30 experiments. Soluble solids, pH and rheological parameters were the selected responses. The authors found that the gums could influence the responses differently. Rheological properties for example, were mostly influenced by the carrageenan while an antagonistic effect of alginate was reported.

Kashaninejad and Razavi [73] used a simplex-centroid design to investigate the effects of hydrocolloids (carboxymethyl cellulose—CMC, konjac gum—KG, and sage seed gum—SSG) on the dynamic rheological properties of instant camel yogurt. A quadratic model was postulated and a total of 14 experiments were carried out which accounted also for the so-called check points. Several models were built, one per each response, and the final optimal conditions were found to be 35% CMC, 10% KG and 55% SSG.

Similar stabilizers (basil seed gum—BSG, CMC and GG) was also studied in ice cream [74]. A simplex-centroid design was set having as responses the apparent viscosity, draw temperature, overrun and melting rate. Ten experiments were carried out although the design was replicated two times considering the total percentage of gum addition as a process variable. This approach is unusual considering that mixture designs are thought to study the effects of proportion of components in the formulation instead of their total amount [6,8,11]. The responses were modeled according to a quadratic model (apparent viscosity and melting rate) and a full quartic model (draw temperature and overrun). The optimum proportions for ice creams with higher viscosity, overrun and melting rate and lower draw temperatures were 84.43% BSG and 15.57% GG at the lowest total concentration. The model was then validated, without any significant differences between the predicted and the measured properties.

Besides the hydrocolloids, the sweeteners have also been optimized [75,76,77]. Indeed, Ribeiro and co-workers used a simplex-lattice design to optimize the sweeteners proportion (three steviol glycoside) in a high-protein plain yogurt. Ten formulations were tested and compared with two reference formulations made by using sucrose and sucralose, respectively. As a response, the Euclidian distance calculated from the Napping^®^ descriptive test was used. The aim was to minimize the distance between the tested formulation and the reference ones. By comparing the contour plots the authors found the optimal ranges of the three components while the optimal formulation was then defined even considering the market price of the single component aiming at the most convenient one.

#### 3.1.4. Case Studies on Juice, Beverages and Jam

Various kind of beverages, drinks and similar products—basically based on fruits—were studied by MD. The effects of lecithin, xanthan gum, propylene glycol alginate, and their combinations on the colloidal stability, the physicochemical and rheological properties of a model peanut-based beverage was studied by Gama et al. [80]. By means of a simplex-centroid design (consisting in seven experiments plus one control), the authors modeled the responses postulating a linear model plus two two-interaction terms. The optimal combination was 66% xanthan gum and 34% lecithin which sum up to 0.5% by weight of the beverage. D-optimal design was used by Shiby et al. [1] to develop a whey-fruit-based energy drink. In this case, two binary mixtures were studied (whey/grape juice and whey/pomegranate juice) having as a response the acidity and the overall acceptability. Quadratic models were postulated after modeling and a mathematical optimization was carried out. Zendeboodi et al. [81] optimized a natural soft drink by studying the effect of whey protein concentrate (WPC), date syrup (DS) and persian gum (PG). A quadratic model was postulated, and nine experiments were carried out. Optimum values of PG, DS and WPC in the mixture design were predicted to be 1.5%, 6.5% and 12%, respectively.

In the work of Kim et al. [26] the aim was to optimize the formulation for antioxidant rich juice powders based on broccoli, cabbage and carrot powders. An augmented simplex-lattice design was used, and the responses were the antioxidant compounds, the antioxidant activity and the acceptance. Linear and quadratic models were calculated respectively for ABTS and acceptance and for total phenolic compounds and FRAP. Finally, the optimization step was carried out. With the aim of optimizing the proportions of black cherry, Concord grape and pomegranate juices in a nutraceutical-rich juice, a simplex-centroid design was set up in Lawless et al. [2]. According to the overall liking and the antioxidant content, the optimal formulation was 75% of Concord grape, 12% pomegranate and 13% black cherry juices.

Interestingly, an augmented simplex-centroid design was used by Dooley et al. [10] to optimize wine blends based on Cabernet Sauvignon, Merlot and Zinfandel to consumers’ liking. The 10 experiments were evaluated by a consumer analysis. Through the desirability function, three blends were created for three distinct targets of consumers.

Aquafaba is an emerging by-product obtained after pulses soaking in water or cooking which presents interesting technological properties [82]. For this reason, it has been used in several practical applications. The main disadvantage is linked to the high moisture content which could be overcome for example by drying. In [82], the optimal formulation of foaming agents and stabilizer to be added to aquafaba prior to the drying process has been studied. A D-optimal design was set up consisting of 25 experiments. The studied components were carboxymethylcellulose, Na-alginate, polydextrose and whey powder, while the responses were foam density and drainage volume. The formulation was numerically optimized obtaining the following mixture: 0.716% CMC, 0.165% Na-alginate, 0.119% polydextrose and 0% whey powder. This mixture, when added to 100 g of aquafaba and dried at 70 °C, gave the best physicochemical features.

Chocolate products have been the focus of several optimization efforts. A simplex-centroid mixture design was used to investigate the effects of four different gums (xanthan gum, guar gum, alginate and locust bean gum) on the rheological properties of a prebiotic model instant hot chocolate beverage [4]. A quadratic model was postulated for a total of 15 experiments (Table 2). The formulation was then mathematically optimized according to the consistency index value and the best solution was found to be a binary mixture made of 59% xanthan gum and 41% locust bean gum. Moreover, the effect of three types of cocoa was studied in the formulation of a hot chocolate beverage [84]. An augmented lattice design was set up and a quadratic model was postulated. The responses were the physicochemical, the rheological and the sensory properties. Thanks to the experiments, three optimized formulations for the sensory properties were identified. In the work of Rezende et al. [85], a simplex-centroid design was used for a three-component mixture (cocoa butter, inulin, β-glucan) aiming at the partial replacement of fat with fiber in sucrose-free chocolates. A quadratic model was postulated. The obtained results showed that β-glucan significantly affected the rheological parameters while inulin led to the improvement of the sensory acceptance.

Processed fruits products are worldwide present and consisted of jellies, jams, juices, nectars, and ice creams and related products. These have been the object of several optimization studies. In Tontul et al. [21] a D-optimal mixture design was used to produce an optimized pomegranate pestil by using xanthan gum, locust bean gum and pregelatinized starch (PS). A total of 16 blends were tested looking at the physicochemical parameters, the texture, the bioactive content and the sensory aspects. The mathematical optimization was carried out through the desirability function. Then, the prediction was validated, and the authors did not find any significant difference between the predicted and the actual formulations, which led to an optimized pestil with higher content of bioactive, less browning and improved textural properties than the traditional one.

Hydrocolloid’s formulations based on carboxymethylcellulose, xanthan gum and pectine in a model system recalling jam/jelly-like products was studied by Ozgur et al. [86]. Ten experiments plus three replicates (one at each vertex) were planned. The contour plots showed the effect of the pure components and of their interactions on the physicochemical and rheological properties. Finally, a mathematical optimization through the desirability function was carried out.

A simplex-lattice design was used to study the formulation of a Brazilian Cerrado fruit jam using five components (fruit jenipapo, marolo, murici, soursop and sweet passion fruit) [5]. A quadratic model was postulated having as responses the consumer acceptance for color, appearance, smell, taste and the overall liking for a total of 21 experimental blends tested. It was found that the formulations with better acceptance for all attributes had percentages of jenipapo, and murici, close to zero and the optimum levels to the other factors were approximately 40% of marolo, 35% of soursop and 25% of sweet passion fruit.

### 3.2. Mixture Design for Extraction Applications

Extraction represents a common step in many areas, from analytical chemistry to by-product valorization. There are different kinds of extractions among which some of the most important in the food area are the liquid-liquid extraction and solid-liquid extraction. In these techniques, a liquid mixture is used to extract target compounds from the matrix. Mixture design of experiments could be an efficient tool to define and optimize the extractive mixture. Some applications in Food Science and Technology have regarded the extraction of bioactive compounds [24,95,96,97]. Water, acetone, acetonitrile and ethanol were the components studied in order to optimize the extraction of isoflavones from defatted soy flour using a simplex-centroid design [95]. A total of 15 experiments were carried out. The authors observed that different forms of isoflavones were differently affected by the mixture composition but in general terms, mixture with 50% water or less are the best. However, for specific class of isoflavones the mixture composition should vary, according to the differences in polarity. Water–acetone–ethanol (2:1:1), water–acetone–acetonitrile (2:1:1) and water–acetone (1:1) mixtures were optimal for extraction of malonyl-glycosidic and total forms, glycosidic isoflavones and less polar aglycone forms, respectively. Mixtures of ethanol, acetone and water were also tested for bioactives extraction from *Phoenix dactylifera* L. seeds by using an augmented simplex-centroid design [97]. The mixture design allowed the identification of synergic or antagonistic effect on the phytochemicals extraction and the optimal mixture was found to be made of 22.39%, 37.37% and 40.24% of acetone, ethanol and water, respectively. An interesting approach is the one followed by Li and co-workers [98] who studied the optimal mixture of raw materials (soybeans, peanuts, linseeds and tea seeds) from which a blended oil with ideal fatty acid ratio of 0.27:1:1 (SFA–MUFA–PUFA) could be extracted. The authors used an augmented simplex-centroid design with replicates for a total of 24 experiments. The responses were MUFA–PUFA, SFA–MUFA and oil extraction yield. It was found that three possible mixtures allowed the authors to reach the goal.

Finally, an augmented simplex-centroid design was also used to define the optimal extraction mixture for the determination of non-target migrants and dibutyl phthalates from baby bottles [99]. The optimized mixture was made up of ethyl acetate (27.5%), dichloromethane (22.5%) and hexane (50%). The results were validated showing a good concordance between the predicted values and the experimental ones.

### 3.3. Mixture Design for the Study of Shelf-Life and Stability

Food degradation could follow different pathways which could vary according to the specific composition of a product, the storage conditions (of both raw materials, ingredients and final product) and the undergone technological processes. In the case of oils and fats, oxidation is of primary concern. Efforts to increase oils stability toward the use of mixture designs have been reported [100,101]. Saoudi and co-workers [100] used an augmented simplex-centroid design to optimize the formulation of natural antioxidants (carnosol, rosmarinic acid and thymol) aimed at increasing soybean oil stability. The ternary combination in equal proportion of the natural antioxidants was more effective in maintaining oxidative stability during heating. In [101] a simplex-lattice design was used to formulate frying oils (from soybean, safflower and flaxseed oils) with high content of essential fatty acids and low *n*-6/*n*-3 ratio. Other studies have regarded the shelf-life of cakes and candies [23,102]. In [102], jujube flour was used to improve the shelf-life of sponge cakes and the results showed that using about 7% of the ingredient in partial substitution of sugar and refined wheat flour improved not only the shelf life of the product but even the textural and physicochemical properties. Spanemberg and colleagues [23] used a D-optimal design to determine the sugar formulation that maximizes the shelf life and critical moisture content of hard candy. In this case, the critical factor affecting the shelf-life was moisture. The optimal formulation of sugars was found to be 0.6, 0.2 and 0.2 of sucrose, 40 DE corn syrup and 20 DE high-maltose corn syrup, respectively, which gave a shelf life that extended seven months beyond the expiration date usually used in the industry.

In the last decades, nanotechnology is spreading also in the food sector and most application regards the effect of nanoparticles in improving stability of dispersed systems. In this regard, the stability of nano emulsions/nano dispersions have been studied using mixture designs [25,103]. Maher and co-workers [103] used a D-optimal design to optimize the formulation of nano emulsions using viscosities and glass transition temperatures as responses. The studied mixture components were lactose, trehalose and β-casein. In [25], Tween 80, gelatin and pectin were the ingredients studied in order to obtain nanoparticles having smallest particle size and highest β-carotene concentrations. The authors set an augmented simplex-centroid design with 10 experiments. The optimal formulation obtained—made of 35% *w*/*w* Tween 80, 46% *w*/*w* gelatin and 19% *w*/*w* pectin—was validated showing that no significant difference were obtained between the predicted and measured responses.

### 3.4. Applications on Food Microbiology

Mixture design was also successfully applied in research involving the microbiological aspects of the food sector. Belay et al. [9] optimized the composition of the atmosphere to reduce the microbial growth during the storage of pomegranate arils by using a simplex lattice mixture design with three components (O_2_, CO_2_, N_2_) and seven experiments. The responses evaluated were the total aerobic mesophilic bacteria, the yeast and the mold counts. The results were fitted to a linear model and then to a special cubic model. The authors found that the single, binary and tertiary component interactions had significant effects in reducing the aerobic mesophilic bacteria and yeast growth. By contrast, the mold count was only significantly affected by the binary interaction of O_2_ and CO_2_. The model was validated, and the special cubic model showed the best predictability; moreover, analyzing the contour plots, the optimum gas concentration was identified.

Yolmeh et al. [104] optimized a multiple-strain mixture (MSM) of *Lactobacillus* against food-borne pathogenic bacteria (*Escherichia coli* (ATCC 25922), *Salmonella enteritidis* (ATCC13076), *Listeria monocytogenes* (ATCC 49594) and *Bacillus cereus* (ATCC 70876)). The cell-free supernatant of *L. brevis* isolated from fermented olives, *L plantarum* isolated from fermented olives and *L. brevis* isolated from garlic, were mixed according to a simplex lattice design and the 14 experiments were tested against the pathogenic bacteria. The antimicrobial activity was explained by a linear model for *E. coli*, *S. enteritidis* and *L. monocytogenes*, whereas for *L. brevis* a quadratic model was significant. Finally, the optimization was carried out by the desirability function, and the accuracy of the prediction was verified.

The antimicrobial activity was studied also by Falleh et al. [105] who optimized the combination of clove, cinnamon, lavender and myrtle essential oils against *E. coli* ATTC 35218, by a simplex centroid design with 15 experiments. The response was modeled by a cubic model. In the optimization step, the authors found that the combination with higher percentages of cinnamon and lavender led to a stronger antimicrobial activity. The optimized formulation was produced, and it showed a similar value compared to the predicted value.

Mahdhi et al. [106] worked on two dates extracts (*Phoenix dactylifera* L.) in combination with a prebiotic *Bacillus* strain to confer protection against vibriosis in Artemia culture. A total of 10 experiments were performed, analyzing the growth rate and the survival rate of the Artemia. The responses were modeled with linear and quadratic equations, the latter showing better results in terms of fitting quality. The authors concluded that the highest protection against virulent *Vibrio* was obtained when only the dates extracts were used, without the combination with *Bacillus*.

Finally, de Castro et al. [107] used a simplex centroid design with seven experiments to investigate the functional properties and the growth promotion of *Bifidobacteria* and lactic acid bacteria strains as affected by protein hydrolysates mixes prepared with soy protein isolate, bovine whey protein and egg white protein. Quadratic or special cubic regression models were fitted with the responses. The model was validated, and the authors did not find any significant difference between the predicted values and the experimental results. The authors concluded that the hydrolyzed samples positively stimulated the growth of the bacteria under investigation, identifying the optimal proportion of the ingredients.

### 3.5. Applications on Food Engineering, Packaging and Related Topics

Within the food engineering area, mixture designs have been successfully applied to develop different kind of films and improve the encapsulation process [3,27,108,109,110]. Pelissari et al. [108] studied the formulation of a film made from cassava starch, chitosan and glycerol. The authors, thanks to the 11 experiments, observed that synergic and antagonistic effects between the components could significantly vary the properties of the film. In particular, chitosan percentage should be controlled in order to avoid excessive rigidity and opacity. Starch based films—using cassava starch, alginate and polyvinyl alcohol—were also studied in [3] by a simplex-lattice design. The best mechanical and barrier properties were found using 80% starch, 11.4% alginate and 8.6% polyvinyl alcohol. In recent years, encapsulation technologies gained attention. Some works have focused on the optimization of wall materials by means of mixture designs for encapsulation of grape seed extract [110], cinnamon essential oil [27] and freeze-dried pumpkin seed oil [109]. In [27,110], augmented simplex-centroid designs allowed the authors to find the optimal formulations which were based on a binary mixture of zein and mesquite gum or maltodextrin [110] and on a ternary mixture of arabic gum, maltodextrin and inulin [27].

### 3.6. Criticism

MD are efficient and rational tools for studying formulation problems which are so common in the food sector. However, from the literature survey, some issues concerning their use have been found.

First, according to Marrubini et al. [18], critical information about the experimental design should be always reported to make the experiments replicable and consequently to verify the results. Unfortunately, the information reported are not always complete. For example, in some cases it is not reported the type of design used (Figure 3B) or even the mathematical model postulated. Lack of information might be linked to the limited space available in scientific articles or to the fact that a secondary importance is given to the explanation of design methodology. Nonetheless, presenting detailed information about the followed design and the model(s) computation could strongly improve the understanding of the results and their practical importance.

Then, a very important concern regards the model coefficients. As previously reported, in mixture designs coefficients do not provide a straightforward understanding of the system under study and the best way to look at the effects of the ingredients/components is by using the surface plots [8,11,28]. Data discussion could be improved if based on contour plots instead of on coefficients and the results could be even more easily understood by the users. A further concern regards the elimination of non-significant coefficients from the postulated models. It could be useful to recall that deleting coefficients from the postulated models has very relevant side effects on the calculations, by increasing the number of degrees of freedom, reducing the leverage and therefore artificially reducing the confidence interval of the predictions. Unfortunately, in many courses the teachers state that the removal of the non-significant coefficients is “the first thing to do”, because in such way “the quality of the model is improved”; of course, they do not realize that this apparent improvement is obtained just by overfitting the data. The best and simple way to face with such issue it to a priori postulate a model and, of course, validate it. If the model is not validated, then it will be possible to postulate another mathematical relationship, calculate the model and, as always, validate it [6]. Another critical aspect regards the use of R^2^ as a figure of merit for evaluating the prediction ability of the models. Indeed, from the literature overview it emerged that, quite often, the suitability of the obtained models for prediction purposes and further real application was based on the high value of R^2^. Thus, it could be useful to recall that the coefficient of determination basically gives some information about the fitting ability. Analysis of variance of the regression, lack of fit test and residuals analysis would be much more useful for checking the model adequacy while a validation step would be the best way for understanding the real applicability of the obtained models.

## 4. Conclusions

This review showed the potentiality of the mixture design as applied in Food Science and Technology. Overall, mixture design is a very versatile tool to investigate a wide range of applications in the food sector, from food technology to microbiology and nutraceutical fields. In the last decade mixture designs have been fairly used in the field of Food Science and Technology, which is ranked fourth among the total records considered for this review work. However, the majority of the application has regarded limited classes of products such as bakery, pasta, juice, beverage, jam and meat. Accordingly, the main ingredients studied have been hydrocolloids, starch and proteins, which are widely used in those products for several technological purposes. Thus, an increase in the spectrum of applications is wished. Interesting areas in which the use of such approach could be useful are for example that of extraction and recovery of bioactives from foods by-products, microbiology or packaging.

The advantage of using mixture designs consists in the possibility to have, with a defined number of experiments, a clear representation of the effect and the interaction of the ingredients in a certain product. Thus, a global knowledge about the system under study could be achieved. Moreover, as reported in most of the examined papers, mixture design allows to split the research and development steps, carrying out, for example, firstly the optimization of the physicochemical or textural properties and then the evaluation of the sensory quality of foods. Only in 14% of the surveyed articles did the number of experiments exceed 20 with clear advantages for the resources management, too.

A main limitation is the difficulty in studying more than three or four ingredients. Perhaps that is why roughly 90% of the surveyed articles consider three components. Finally, some criticisms have been found and highlighted, basically linked to the presentation of clear information about the model definition and development, the coefficients interpretation and the model validation.

On the whole, we strongly encourage the researchers to implement the use of mixture design for their works, in order to improve the reliability of the results as well as to optimize the work and the resources.

## Figures and Tables

**Table 1:** Experimental points (blends of the components *X*1, *X*2, *X*3) for a special cubic model according to the simplex-lattice or the simplex-centroid designs.

#	Simplex-Lattice Design			Simplex-Centroid Design		
	*X*1	*X*2	*X*3	*X*1	*X*2	*X*3
1	1	0	0	1	0	0
2	0	1	0	0	1	0
3	0	0	1	0	0	1
4	2/3	1/3	0	0.5	0.5	0
5	2/3	0	1/3	0.5	0	0.5
6	1/3	2/3	0	0	0.5	0.5
7	0	2/3	1/3	1/3	1/3	1/3
8	1/3	0	2/3	-	-	-
9	0	1/3	2/3	-	-	-
10	1/3	1/3	1/3	-	-	-

**Table 2:** Number of experiments for simplex-lattice and simplex-centroid designs according to the number of components (q) and the postulated models.

q	Simplex-Lattice Design			Simplex-Centroid Design
	Linear	Quadratic	Special Cubic	
2	2	3	4	3
3	3	6	10	7
4	4	10	20	15
5	5	15	35	31
6	6	21	56	63
7	7	28	84	127

**Table 3:** Number of results divided in subject area for the keyword “mixture-design”.

Web of Science Categories	Records	% of 2169
Materials Science Multidisciplinary	507	23
Engineering Civil	488	22
Construction Building Technology	454	21
Food Science Technology	330	15
Pharmacology Pharmacy	174	8
Engineering Chemical	167	8
Environmental Sciences	121	6
Biotechnology Applied Microbiology	90	4
Engineering Environmental	90	4
Energy Fuels	89	4

Results obtained from Web of Science database. Each record can belong to more than one category.

**Table 4:** Summary of the main features of the mixture designs applied together with the area of investigation.

Reference	Food Product/Application	Components	No. Components	Model ^a^	Design	No. Experiments ^b^	Model Fitting ^c^	Validation ^d^	Surface Plots ^e^	Optimization ^f^
*Bakery Products and Pasta*										
[34]	Bread	Hydroxypropyl methyl cellulose, xanthan and guar gums	3	Quadratic	D-optimal	11	Anova of regression, R^2^, R^2^ adj, R^2^ pred, Lack of fit, PRESS	No	Yes	Yes
[35]	Bread	Rice flour, maize starch and wheat starch	3	Special cubic	Simplex-centroid	7	Anova of regression, R^2^	No	Yes	Yes
[36]	Quick breads (scones)	Margarine, oligofructose, caster sugar and inulin	4	Special cubic	D-optimal	24	Anova of regression, R^2^	Yes	Yes	Yes
[37]	Bread	Chickpea flour, cassava starch, maize starch, potato starch and rice flour	3	Linear, quadratic, special cubic	Simplex-centroid	9 (×6 designs)	Anova of regression, R^2^ adj, Lack of fit	Yes	Yes	Yes
[38]	Gluten-free sponge cake	Whey protein concentrate, maize and rice flours	3	Linear, quadratic, special cubic	Not reported	15	Anova of regression, R^2^, R^2^ adj	No	No	Yes
[39]	Sponge cake	Powdered nettle leaf and milk thistle	2	Linear, quadratic, cubic, quartic	Not reported	9	Anova of regression, R^2^, R^2^ adj	Yes	Yes	Yes
[40]	Muffin	Xathan, carboxymethylcellulose and κ-carrageenan	3	Linear, quadratic, special cubic and cubic	Augmented simplex-centroid	10	R^2^, R^2^ pred	No	Yes	Yes
[41]	Gluten-free layer cakes	Pea, whey and egg white proteins	3	Cubic	Simplex-lattice	10	R^2^	No	Yes	No
[42]	Cake	Chhana, tikhur starch and semolina	3	Linear, quadratic, special cubic, cubic	D-optimal	14	R^2^, R^2^ adj, Lack of fit, PRESS	Yes	Yes	Yes
[20]	Pasta	Water, proteins and gums	3	Quadratic	Augmented simplex-centroid	13	Anova of regression, Lack of fit	No	Yes	Yes
[43]	Rice noodle	Gelatinized corn starch, guar gum and xanthan gum	3	Special cubic	Simplex-centroid	7	R^2^	No	Yes	Yes
[44]	Pasta	Semolina, pea protein isolate 80%, whey protein isolate 80%, soy protein isolate 90%, oat flour and gluten	6	Linear, quadratic and special cubic	D-optimal	31	R^2^ pred, Lack of fit, Cook’s distance, DFFITS	No	No	Yes
[45]	Bread	Xanthan gum, sodium alginate and guar gum	3	Quadratic, cubic	Simplex-centroid	7	R^2^	No	Yes	Yes
[46]	Dough	Einkorn, cranberry bean and potato flours	3	Quadratic	Simplex-lattice	15	Anova of regression, R^2^, RMSE	No	No	No
[47]	Cake	Wheat flour, yacon flour and maca flour	3	Special cubic	Simplex-centroid	7 (21 considering the mixture-process design)	Anova of regression, R^2^ adj, Relative error	No	Yes	Yes
[48]	Crackers	Cassava starch, high quality cassava flour and fish flour	3	Quadratic, special cubic, cubic	Simplex-centroid	14	R^2^	No	Yes	Yes
[49]	Breadmaking improvers	*Aspergillus oryzae* S2 α-amylase, ascorbic acid and glucoseoxidase	3	Special cubic	Simplex-centroid	7	R^2^	Yes	Yes	Yes
[50]	Cookies	Wheat flour, Jerusalem artichoke flour and sugar	3	Quadratic	Simplex-centroid	7	R^2^	No	Yes	Yes
[51]	Cookies	Quinoa flour, quinoa flakes and corn starch	3	Cubic	Simplex-lattice	14	R^2^, R^2^ adj	No	Yes	Yes
[52]	Aqueous model system	Wheat, buckwheat and rice flours	3	Linear, quadratic	Simplex-lattice	14	R^2^	No	Yes	No
[53]	Wheat chips	Chickpea flour, pea flour and soy flour	3	Quadratic	Simplex-lattice	15	Anova of regression, R^2^, RMSE	No	Yes	Yes
[19]	Extruded corn snacks	Fish protein, cheddar cheese powder and vegetable oil; omega-3 fish oil, vegetable oil and cheese powder	3	Not reported	D-optimal	14	Not reported	No	Yes	Yes
[54]	Dough	Wheat flour, WF, Lupinus protein concentrate and Jatropha protein concentrate	3	Special cubic	D-optimal	8	Anova of regression, R^2^	Yes	Yes	Yes
[55]	Extruded product	Maize, finger millet and defatted soy flours	3	Linear, quadratic, cubic, special cubic	D-optimal	16	Anova of regression, R^2^, R^2^ adj, Lack of fit	Yes	Yes	Yes
*Meat products*										
[56]	Beef burgers	Inulin, β-glucan and breadcrumbs	3	Linear, quadratic, special cubic	D-optimal	14	R^2^	Yes	Yes	Yes
[57]	Sausage	Pork back fat, inulin (two types)	3	Linear, quadratic, special cubic	D-optimal	17	Anova of regression, R^2^, Lack of fit	No	Yes	Yes
[58]	Sausage	Whey powder, ι-carrageenan and turkey fat	3	Special cubic	Simplex-lattice	14	R^2^, R^2^ adj	No	Yes	Yes
[59]	Sausage	Inulin, konjac and starch	3	Linear, quadratic, special cubic	D-optimal	13	R^2^ pred	No	Yes	Yes
[60]	Sausage	β -glucan, resistant starch and starch	3	Linear, quadratic, special cubic	D-optimal	13	R^2^ pred	No	No	Yes
[61]	Sausage	*Persicaria hydropiper*, *Murraya koenigii* and *Etlingera elatior* dried aromatic herbs	3	Linear, quadratic, special cubic	Simplex-lattice	13	Anova of regression, R^2^, Lack of fit	Yes	Yes	Yes
[62]	Sausage	NaCl, KCl and sodium tripolyphosphate	3	Cubic	Augmented simplex-centroid	13	Anova of regression, Lack of fit	Yes	Yes	Yes
[63]	Beef patties	Meat, lentil flour and rice protein	3	Linear, quadratic, special cubic	D-optimal	17	Anova of regression, R^2^, Lack of fit	Yes	Yes	Yes
[64]	Beef patties	Pork fat, pomace olive oil and canola oil	3	Special cubic	Augmented simplex-centroid	10	R^2^ adj	No	Yes	No
[65]	Ham	NaCl, glycine and yeast extract	3	Linear, quadratic	I-optimal	12	Anova of regression, R^2^ adj, Lack of fit	Yes	Yes	Yes
[66]	Canned meat	Pork bacon, inulin and lentil flour	3	Special cubic	Simplex-centroid	10	R^2^	No	Yes	No
[19]	Fish strudel	Fish mince, onion and curry powder	3	Not reported	D-optimal	14	Not reported	No	Yes	Yes
[67]	Lamb patties	Fat, carboxymethyl cellulose and inulin	3	Special cubic	Simplex-lattice	13	Not reported	No	Yes	No
[68]	Beef patties	Grape seed oil, pomegranate seed oil and animal fat	3	Quadratic	Simplex-lattice	15	R^2^	No	No	No
*Emulsions, creams, desserts, juices, beverages, jams*										
[69]	Mayonnaise-like product	Xanthan gum, guar gum and mono-diglycerides emulsifier	3	Linear, qudratic, special cubic	Augmented simplex-centroid	10	R^2^	No	Yes	Yes
[70]	Mayonnaise	Germ protein isolate, xanthan gum and egg yolk	3	Linear, quadratic, special cubic	Undefined optimal design	10	R^2^	No	Yes	Yes
[71]	Dairy dessert	Carrageenan, alginate, guar and xanthan gums	4	Cubic	Simplex-lattice	30	R^2^	No	Yes	Yes
[72]	Dairy dessert	*A. gossypinus*, *A. fluccosus* and *A. rahensis tragacanth gums*	3	Linear, quadratic, special cubic, cubic	Not reported	17	R^2^	No	No	No
[73]	Camel yogurt	Carboxymethyl cellulose, konjac gum and sage seed gum	3	Quadratic	Simplex-centroid	14	Anova of regression analysis, R^2^, Lack of fit	No	Yes	Yes
[74]	Ice cream	Basil seed gum, carboxymethyl cellulose and guar gum	3	Quadratic, full quartic	Augmented simplex-centroid	10 (x2 different total amount)	Anova of regression, R^2^, R^2^ adj	Yes	Yes	Yes
[75]	Yogurt	Steviol glycoside sweeteners	3	Linear, special cubic	Simplex-lattice	10	Anova of regression, R^2^	No	Yes	Yes
[76]	Sweeteners blend	Coconut sugar, agave and stevia	3	Linear, quadratic, special cubic	Augmented simplex-centroid	10	R^2^ adj	No	Yes	Yes
[77]	Milk chocolate	Maltitol, xylitol and isomalt	3	Linear, quadratic, special cubic	Simplex-lattice	15	Anova of regression, R^2^, R^2^ adj, Lack of fit	Yes	Yes	Yes
[78]	Chocolate	Maltitol, xylitol and galactooligosaccharide	3	Linear, quadratic	Simplex-lattice	14	Anova of regression, R^2^, R^2^ adj, Lack of fit	Yes	Yes	Yes
[79]	Spreadable halva	Soy flour, sesame paste and emulsifier	3	Linear, quadratic, special quartic	Undefined optimal design	12	Anova of regression, R^2^	No	No	Yes
[80]	Peanut-based beverage	Lecithin, xanthan gum, propylene glycol alginate	3	Linear plus two interaction terms	Simplex-centroid	7	R^2^, R^2^ adj, RMSE	No	Yes	Yes
[1]	Energy drink	Whey and grape juice; whey and pomegranate juice	2	Quadratic	D-optimal	13	R^2^	Yes	Yes	Yes
[81]	Soft drink	Whey protein concentrate, date syrup and persian gum	3	Quadratic	Not reported	9	R^2^	No	Yes	Yes
[26]	Juice powders	Broccoli, cabbage and carrot powders	3	Linear, quadratic	Augmented simplex-lattice	13	Anova of regression, R^2^	No	Yes	Yes
[2]	Juice	Black cherry, Concord grape and pomegranate juices	3	Not reported	Simplex-centroid	7	Not reported	No	Yes	Yes
[10]	Wine blends	Cabernet Sauvignon, Merlot and Zinfandel	3	Linear	Augmented simplex-centroid	10	Not reported	Yes	Yes	Yes
[82]	Aquafaba	Carboxymethylcellulose, Na-alginate, polydextrose and whey powder	4	Quadratic	D-optimal	25	Anova, R^2^, R^2^ adj, Lack of fit, PRESS	No	Yes	Yes
[83]	Chocolate	Xanthan gum, guar gum, alginate and locust bean gum	4	Quadratic	Simplex-centroid	15	R^2^	No	Yes	Yes
[84]	Chocolate	Usta cocoa, Gerkens cocoa and Ulker Gold cocoa	3	Quadratic	Augmented simplex-lattice	9	R^2^	No	Yes	Yes
[85]	Chocolate	Cocoa butter, inulin and β-glucan	3	Quadratic	Simplex-centroid	8	Anova of regression, R^2^ adj, Lack of fit	No	Yes	No
[21]	Pestil (fruit leather)	Xanathan gum, locust bean gum and pregelitinized starch	3	Linear, quadratic, special cubic, cubic, quartic and special quartic	D-optimal	16	R^2^, Lack of fit	Yes	Yes	Yes
[86]	Jam/jelly-like model system	Xanthan gum, pectin and carboxymethyl cellulose	3	Not reported	Simplex-lattice	13	R^2^	No	Yes	Yes
[5]	Jam	Jenipapo, marolo, murici, soursop and sweet passion fruit pulps	5	Quadratic	Simplex-lattice	21	Anova of regression, R^2^	Yes	Yes	Yes
[22]	Tomato sauce	Hot-break tomato puree, onion puree and extra virgin olive oil; Cold-break tomato puree, onion puree and extra virgin olive oil	3	Linear, quadratic, special cubic	D-optimal	14	Anova of regression, R^2^, R^2^ adj, Lack of fit	No	Yes	No
[87]	Berry jelly	Blackberry, blueberry and strawberry fruit juices	3	Linear, quadratic	Simplex-centroid	7	Anova of regression, R^2^, Lack of fit	No	Yes	Yes
[88]	Fruit jelly	Jabuticaba, pitanga and cambuci fruit juices	3	Linear	Simplex-centroid	7	R^2^, Lack of fit	No	Yes	Yes
[89]	Soy-based sauce	Guar gum, xanthan gum and pregelatinized cassava starch	3	Linear, quadratic, cubic	Simplex-centroid	9	R^2^, Lack of fit	No	Yes	Yes
[90]	Instant ice cream mix powder	Carboxymethyl cellulose, carrageenan and sodium alginate	3	Cubic	D-optimal	14	R^2^, R^2^ adj	No	Yes	Yes
[91]	Beverage	Sweet potato peel water extract, sweet potato leaves water extract and honey solution	3	Cubic	I-optimal	24	Anova of regression, R^2^, R^2^ adj, Lack of fit, RMSE	No	Yes	Yes
[83]	Dairy dessert	Carrageenan, alginate, guar and xanthan gums	4	Reduced cubic, reduced special cubic, reduced special quartic	Simplex-lattice	30	R^2^	No	Yes	Yes
[92]	Soy-based fermented product	Soy, oat and wheat fibers	3	Linear, quadratic, special cubic	Simplex-centroid	9	R^2^, Lack of fit	No	Yes	Yes
[93]	*In vitro* flavor release	Maltitol, erythritol, polydextrose and oligofructose	4	Quadratic	D-optimal	20	R^2^	No	Yes	No
[53]	Beverage	Pine, flower and highland honeys	3	Quadratic	Simplex-lattice	15	R^2^	No	Yes	Yes
[94]	Salad dressing	Chinese quince juice, oil and vinegard	3	Linear, quadratic	D-optimal	14	Anova of regression, R^2^	No	Yes	Yes
*Extraction applications*										
[95]	Isoflavone extraction from soy flour	Water, acetone, acetonitrile and ethanol	4	Linear, quadratic, special cubic	Simplex-centroid	15	Anova of regression, R^2^, Lack of fit	Yes	Yes	Yes
[96]	Bioactive extraction from fruits	Acetone, methanol and water	3	Quadratic	Simplex-lattice	15	Anova of regression, R^2^, R^2^ adj, R^2^ pred, Lack of fit	Yes	Yes	Yes
[97]	Bioactive extraction from seeds	Ethanol, acetone and water	3	Linear, quadratic, special cubic	Augmented simplex-centroid	10	Anova of regression, R^2^	No	Yes	Yes
[24]	Extraction from herbs	*Rhodiola crenulata*, *Panax quinquefolius* and *Astragalus membranaceus*	3	Linear, quadratic	Augmented simplex-centroid	10	Anova of regression, R^2^, R^2^ adj	No	Yes	Yes
[98]	Oil extraction	Soybeans, peanuts, linseeds and tea seeds	4	Linear, quadratic, cubic, special cubic	Augmented simplex centroid	24	Anova of regression, R^2^, R^2^ adj, R^2^ pred, Lack of fit	Yes	Yes	Yes
[99]	Migration from baby bottles	Toluene–hexane, dichloromethane–hexane and ethyl acetate–hexane	3	Linear, quadratic, cubic	Augmented simplex-centroid	13	Anova of regression, Lack of fit	Yes	No	Yes
*Study of shelf-life and stability*										
[100]	Stability of soybean oil	Carnosol, rosmarinic acid and thymol	3	Special cubic	Augmented simplex centroid	10	R^2^	No	Yes	No
[101]	Frying oil	Soybean, safflower and flaxseed oils	3	Linear, cubic	Simplex-lattice	185	Anova of regression	Yes	No	Yes
[102]	Shelf life of sponge cake	Refined wheat flour, sugar, jujube fruit flour	3	Linear, quadratic, special cubic	Undefined optimal design	16	Anova of regression, R^2^, R^2^ adj, R^2^ pred, Lack of fit	Yes	Yes	Yes
[23]	Shelf life of hard candy	Sucrose, 40 DE corn syrup and 20 DE high-maltose corn syrup	3	Linear, quadratic, cubic	D-optimal	36	Anova of regression, R^2^, R^2^ adj, Lack of fit, residuals analysis	Yes	Yes	Yes
[103]	Nanoemulsion	Lactose, trehalose and β-casein	3	Quadratic, special cubic, cubic	D-optimal	17	Anova of regression, R^2^ adj	No	Yes	Yes
[25]	β-carotene nanoparticles	Tween 80, gelatine and pectin	3	Special cubic	Augmented simplex-centroid	10	Anova of regression, R^2^	Yes	Yes	Yes
[9]	Gas mixture	O_2_, CO_2_, N_2_	3	Linear, special cubic	Simplex-lattice	7	R^2^	Yes	Yes	Yes
*Food microbiology*										
[104]	Microbiology	Cell-free supernatants of *L. brevis* and *L. plantarum*	3	Linear, quadratic, special cubic, cubic	Simplex-centroid	14	Anova of regression, R^2^, R^2^ adj, R^2^ pred, Lack of fit	Yes	Yes	Yes
[105]	Antimicrobial activity	Clove, cinnamon, lavender and myrtle essential oils	4	Cubic	Simplex-centroid	15	Anova of regression, Residuals	Yes	Yes	Yes
[106]	Microbiology	Deglet Nour extract, Deglat extract and *Bacillus* strain	3	Linear, quadratic	D-optimal	10	Anova of regression, Lack of fit	No	Yes	Yes
[107]	Microbiology	Soy protein isolate, bovine whey protein and egg white protein	3	Quadratic, special cubic	Simplex-centroid	7	Anova of regression, R^2^ adj	Yes	Yes	No
*Food engineering, packaging and related topics*										
[108]	Film production by blown extrusion	Cassava starch, chitosan and glycerol	3	Special cubic	Not reported	11	R^2^, Lack of fit	No	Yes	No
[3]	Starch-based films	Cassava starch, alginate and polyvinyl alcohol	3	Cubic	Augmented simplex-lattice	10	Anova of regression, R^2^	No	Yes	Yes
[109]	Microencapsulation	Whey protein concentrate, maltodextrin and arabic gum	3	Linear, reduced quadratic, quadratic, reduced cubic, special cubic, reduced special quartic, special quartic	D-optimal	18	Anova of regression, R^2^	No	Yes	Yes
[27]	Microencapsulation	Arabic gum, maltodextrin and inulin	3	Cubic	Augmented simplex-centroid	16	Anova of regression, R^2^	Yes	Yes	Yes
[110]	Extract microencapsulation	Maltodextrin, mesquite gum and zein	3	Not reported	Augmented simplex-centroid	10	Not reported	No	No	Yes
[111]	Salt reduction	NaCl, KCl and CaCl_2_	3	Linear, quadratic, special cubic	Not reported	10	R^2^ adj	No	Yes	Yes

^a^, postulated and/or fitted canonical models. Reduced models obtained after coefficient(s) removing are not reported except if explicitly indicated in the text; ^b^, considering also replicates; ^c^, figure of merits of the goodness of fit shown; ^d^, validation of the fitted model by test experiments; ^e^, response(s) contour plot(s); ^f^, defining an optimal formulation by numerical or graphical approaches.

## Data Availability

Not applicable.
